# Mediating effect of illness perception between self-care ability and health-promoting behaviors among patients with stable coronary artery disease

**DOI:** 10.1371/journal.pone.0316551

**Published:** 2025-02-21

**Authors:** Yuanyuan Wang, Xinyue Gong, Jing Cheng, Yingting Wu, Sihan Wang, Ying Zhu, Changyi Liu, Fei He, Kehui Xu

**Affiliations:** 1 School of Nursing, Anhui University of Chinese Medicine, Hefei, China; 2 Department of Cardiology, The Second Affiliated Hospital of Anhui Medical University, Hefei, China; 3 The Second Affiliated Hospital of Anhui University of Chinese Medicine, Hefei, China; Children’s Hospital of Eastern Ontario (CHEO), University of Ontario, CANADA

## Abstract

**Background:**

The interaction between illness perception, self-care ability, and health-promoting behaviors (HPB) in stable coronary artery disease (SCAD) patients remains uncertain. We conducted a cross-sectional survey to explore the correlation between self-care ability, illness perception, and HPB among patients with SCAD, as well as the potential mediating role of illness perception between self-care ability and HPB.

**Methods:**

A cross-sectional study was carried out among 184 inpatients with SCAD in Hefei, China, from December 2022 to March 2023. The Self-Care of Coronary Heart Disease Inventory (SC-CHDI, containing three dimensions: self-care maintenance, self-care management, and self-care confidence), Revised Illness Perception Questionnaire (IPQ-R, containing seven dimensions: illness duration, illness consequence, personal control, treatment control, illness coherence, cyclical timeline, emotional distress), Health-Promoting Lifestyle Profile Ⅱ (HPLP-Ⅱ) were used in the questionnaires. SPSS 25.0 software and PROCESS version 4.2 plug-in was used to analyze the mediating effect.

**Results:**

HPB of SCAD patients was at moderate level. A range of factors including education level, marital status, self-care maintenance, self-care management, self-care confidence, illness coherence, and emotional distress are potential influencers of HPB. Illness coherence had a partially mediated effect between self-care maintenance and HPB (*β* = 0.063, 95% CI: 0.021~0.111), accounting for 20.59% of the total effect. Similarly, illness coherence had a partially mediated effect between self-care management and HPB (*β* = 0.055, 95% CI: 0.016~0.105), accounting for 13.78% of the total effect. However, none of the dimensions of illness perception mediated between self-care confidence and HPB. Self-care confidence directly influenced HPB, accounting for 92.40% of the total effect.

**Conclusion:**

It is necessary for hospital healthcare workers, community workers, and patients’ families to work together to focus on the self-care ability and positive illness perception of patients with cardiovascular disease, so as to increase patients’ motivation to participate in HPB and improve their quality of life.

## Introduction

The global prevalence of cardiovascular disease (CVD) is on the rise due to various factors such as changes in lifestyle, urbanization, and aging. According to WHO, ischaemic heart disease ranked first among the top 10 leading causes of death globally in 2021 [1]. Since 2000, there has been a significant increase in the number of deaths due to ischaemic heart disease worldwide. In China, the *Report on Cardiovascular Health and Diseases in China 2022* indicated a staggering 330 million individuals affected by cardiovascular conditions, with 11.39 million suffering from coronary artery disease (CAD) [2]. Stable coronary artery disease (SCAD, also referred to as chronic coronary syndrome) represents the most prevalent form of CAD and denotes a period of stability following chronic stable exertional angina, ischemic cardiomyopathy, and acute coronary syndrome [3]. SCAD is characterized by a prolonged disease course and is associated with increased mortality, disability, and a high risk of recurrence. These challenges impose significant burdens on healthcare institutions, society, families, and individuals [4]. Relying solely on healthcare institutions and professionals for the management of SCAD in the long term is not sustainable. Research into CVD prevention and risk factor management consistently emphasizes the importance of adopting HPB to enhance cardiovascular risk profiles and potentially alleviate the burden of CVD [5].

Health-promoting behaviors (HPB) refer to voluntary, modifiable actions taken to maintain or improve one’s own health status [6]. However, studies have shown that individuals with CAD often exhibit unhealthy lifestyles, including smoking, excessive alcohol consumption, poor dietary choices, physical inactivity, and unfavorable psychological habits [7–9]. These behaviors pose a latent risk for CAD recurrence and hospital readmission. The cultivation of a healthy lifestyle is intricately linked to the risk profile of cardiovascular disease [10].

**Fig 1:**
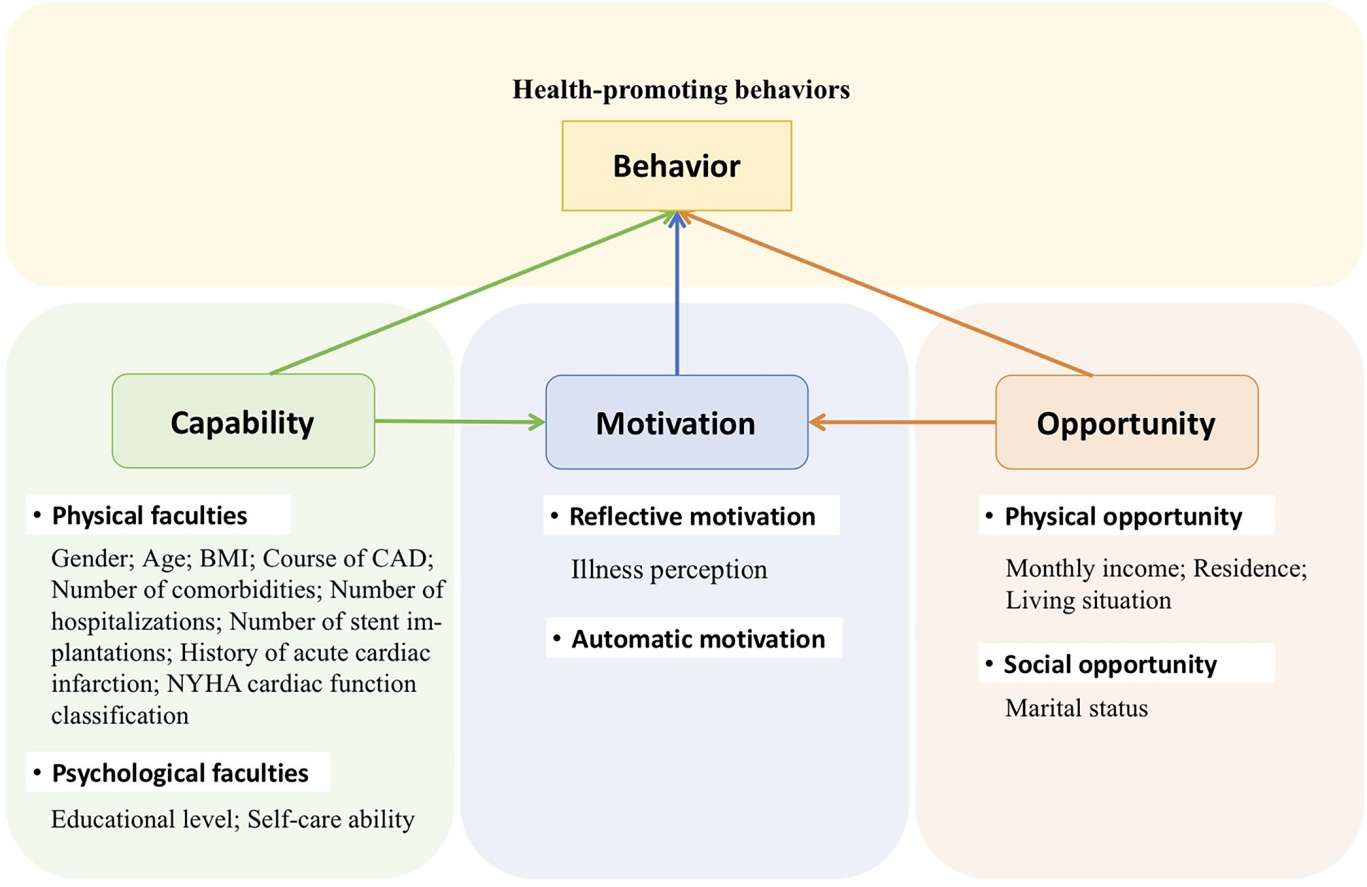
Theoretical framework diagram of this study based on COM-B model.

We applied the COM-B (capability, opportunity, motivation to perform a behavior) [11] theoretical model to guide the selection of variables affecting HPB in SCAD patients (Fig 1). This theory posits that an individual’s behavior is influenced by three factors: capability, opportunity and motivation. Capability refers to the physical and mental faculties that enable an individual to perform relevant actions [11]. Self-care refers to the management of disease risk factors by patients themselves. It has been proven effective in preventing, delaying, and controlling cardiovascular diseases, and is crucial for reducing their incidence and prevalence [12]. However, overall self-care ability among patients with cardiovascular disease is often poor [13]. Previous research has shown that improving self-care ability can lead to better disease prognosis, reduced psychological distress, improved quality of life, and increased adoption of HPB [14, 15].

Motivation encompasses reflective motivation (conscious intentions, plans, and evaluations) and automatic motivation (emotional reactions, impulses, and desires) [11]. Individuals have unique understandings and perceptions of their diseases, stemming from personal experiences, interactions with healthcare professionals, and discussions with other patients. This phenomenon is known as illness perception [16]. Illness perception can lead individuals to develop beliefs about behavioral change and can directly influence the willingness of CAD patients to undergo cardiac rehabilitation [17]. Illness perception can influence patients’ reactions to treatments and disease management strategies. Psychological cognitive theories suggest that illness perception acts as a vital mediating variable between cardiac rehabilitation and health behavior, directly impacting individual health outcomes [18]. Several studies have demonstrated that enhancing illness perception can improve patients’ self-care ability, quality of life, and disease prognosis [19].

Opportunities are external factors that facilitate individual behavior, including elements of the physical opportunity (time and resources) and social opportunity (health education courses, support from others). Both capability and opportunity can directly influence the manifestation of behavior, as well as exert an indirect effect on the formation of behavior through motivation [11]. Therefore, in this study, illness perception was selected as a mediating variable between self-care ability and HPB in SCAD patients.

## Materials and methods

### Study design

A cross-sectional survey was performed in this study by using convenience sampling.

### Participants

The participants in this study were recruited from the Department of Cardiology at the Affiliated Hospital of Anhui University of Chinese Medicine in China during their inpatient hospitalization, between December 2022 and March 2023. The inclusion criteria for selecting participants were as follows: (a) meeting the diagnostic criteria for stable coronary artery disease (SCAD) as defined by medical professionals [3]; (b) being in a stable physical condition during the data collection period and showing no cognitive impairments; (c) being 18 years of age or older. On the other hand, the exclusion criteria were: (a) individuals with severe mental or neurological disorders, such as severe stroke or epilepsy; (b) those suffering from severe physical illnesses, including malignant tumors or serious dysfunction in organs such as the liver or kidneys; (c) participants with significant hearing or visual impairments; (d) individuals who were unable to comprehend the purpose of the questionnaire or faced barriers in communication.

### Data collection

This study obtained approval from the Ethics Committee of the Affiliated Hospital of Anhui University of Chinese Medicine (Approval No. IRB FILE 2022-zjmc-04). Prior to the commencement of the study, participants were provided with detailed information regarding the purpose and significance of the research. Researchers conducted face-to-face data collection sessions only after obtaining informed consent from the patients. Upon completion of the questionnaire, the researchers conducted an immediate review of the responses on-site. The questionnaires were centrally administered, systematically reviewed, and assigned unique identification numbers for data organization and analysis purposes.

Following the sample size estimation principle proposed by Kendall M [20], which recommends a sample size of 5 to 10 times the number of variables, this study comprised 14 variables related to personal basic information, 3 variables from the coronary heart disease self-care scale, 7 variables from the illness perception questionnaire, and HPB. Taking into account a potential 10% loss in tracking rate, the minimum required sample size for this study was determined to be 160 participants. Subsequently, a total of 184 participants were enrolled in the study using convenience sampling method, ensuring a robust representation of the target population for comprehensive data analysis and interpretation.

### Measures

#### Sociodemographic questionnaire

By incorporating a comprehensive range of sociodemographic and clinical variables, the questionnaire aimed to capture a holistic view of the participants’ backgrounds and health statuses. The self-designed general information questionnaire utilized in this study comprised two main sections: (a) sociodemographic characteristics, including age, gender, education level, marital status, monthly income, residence, and living situation; (b) clinical information characteristics, encompassing height, weight, New York Heart Association (NYHA) cardiac function classification, disease duration, number of hospitalizations, number of stent implantations and comorbidities, and history of acute cardiac infarction. These details were primarily gathered through patient interviews or by reviewing their medical records. (Patient self-reported variables: educational level, monthly income, marital status, residence, living situation, number of hospitalizations, history of acute cardiac infarction. Objective variables in the medical record: gender, age, body mass index (BMI), course of CAD, number of stent implantations, number of comorbidities, and NYHA cardiac function classification.)

#### Self-Care of Coronary Heart Disease Inventory (SC-CHDI)

The Self-Care of Coronary Heart Disease Inventory (SC-CHDI) was initially developed by Dickson and later translated into Chinese by Chen in 2018 [21, 22]. It serves as an assessment tool to evaluate the self-care behavior of patients with CAD. The scale comprises three subscales which collectively consist of 22 items. The self-care maintenance subscale, comprising 10 items, evaluates the patient’s adherence to the treatment regimen and autonomous practices promoting health. The self-care management subscale, comprising 6 items, evaluates the patient’s recognition of, and response strategies to, changes in symptoms and physical signs. The self-care confidence subscale, encompassing 6 items, evaluates a patient’s confidence in executing self-care maintenance, monitoring, and management. Each subscale is standardized with a maximum score of 100. Scores below 70 indicate inadequate self-care behavior or low self-care confidence. In this study, the Cronbach’s alpha coefficients for each dimension ranged from 0.485 to 0.871.

#### Revised Illness Perception Questionnaire (IPQ-R)

Illness perception was assessed based on the Chinese version of the IPQ-R [23]. The questionnaire consists of 7 dimensions (illness duration, illness consequence, personal control, treatment control, illness coherence, cyclical timeline and emotional distress) and 38 items. Illness duration explores the patient’s perception of the duration of their illness. Illness consequence evaluates the impact of the disease on the patient’s quality of life. Personal control quantifies patients’ convictions about the effect of their behaviors on disease progression. Treatment control assesses the patient’s perceived efficacy of the treatment. Illness coherence investigates the patient’s understanding of their condition. Cyclical timeline assesses the cyclical nature of disease progression in patients. Emotional distress studies the emotional toll the disease takes on the patient [24]. A Likert 5-point scale was used to assign a score of 1 (“completely disagree”) to 5 (“completely agree”) and 13 items reverse scored. Higher scores on personal control, treatment control, and illness coherence indicate higher level of positive perception. And higher scores on the remaining four dimensions indicate higher level of negative perception. The Cronbach’s alpha coefficients for each dimension ranged from 0.597 to 0.911 in this study.

#### Health-Promoting Lifestyle Profile II (HPLP-II)

Health-promoting behavior was evaluated by the HPLP-Ⅱ [25, 26]. The scale consists of six dimensions (health responsibility, nutrition, stress management, exercise, interpersonal relationships, and self-actualization) with 52 items. A 4-point Likert scale (from 1 to 4) was used, ranging from “never” to “always”. A higher total score on the HPLP-II scale represents a better HPB. The scale is divided into four levels: excellent (173–208 points), good (132–172 points), moderate (92–131 points), and bad (52–91 points). The Cronbach’s alpha was 0.91 in this study.

### Statistical analysis

Data were inputted using EpiData 3.1 software, and the collated data were imported into SPSS 25.0 software for analysis, with a test level of two-sided *α* = 0.05. Data for normally distributed continuous variables were described using mean ± standard deviation. Data with a skewed distribution were described by median and interquartile spacing. Data on categorical variables was described using frequency counts and proportions. Between-group comparisons for normally distributed continuous variables utilized either the independent samples t-test or F-test as appropriate. In terms of questionnaire data validity, we used the Harman single-factor analysis for common method bias [27]. Pearson correlation analysis was used to explore the correlation between self-care ability, illness perception and HPB. Linear regression analysis was employed to initially explore the influence of self-care ability and illness perception on HPB. The PROCESS version 4.2 plug-in was used to analyze the mediating effect [28]. The bootstrap method was used to sample 5000 times, and the indirect effects were controlled according to the value of the 95% confidence interval. If the 95% confidence interval does not pass through zero, the mediating (indirect) effect is valid. Two-tailed *P* < 0.05 was considered statistically significant.

## Results

### Sample characteristics

A total of 184 hospitalized patients diagnosed with stable coronary artery disease (SCAD) were enrolled in this study. Of these participants, 63.6% were male. In terms of educational background, 59.8% had received a junior school education or higher. Regarding monthly income, 51.6% reported earning less than 3000 yuan. The survey found that the majority of participants were married (79.3%). Notably, overweight and obese patients accounted for 59.2%. In relation to disease characteristics, 64.7% of participants reported a duration of SCAD of more than 6 months. Additionally, 21.2% had been hospitalized for coronary heart disease (CHD) more than three times. Participants commonly suffered from comorbidities, with 89.7% of patients associated with one or more comorbidities. Notably, 37.5% reported a history of acute coronary syndrome, and 44.6% were classified as NYHA grade I for cardiac function. Further details regarding the demographic and clinical characteristics of the participants can be found in Table 1. To screen for common method bias, we employed Harman single-factor analysis. The results revealed that the interpretation rate of the unrotated first factor was 12.607% (less than the critical criterion of 40%). Therefore, there was no significant common method bias in this study.

**Table 1:** Sociodemographic and clinical characteristics of SCAD patients (*n* = 184).

Characteristics	Project	*n* (*%*)	Characteristics	Project	*n* (*%*)
Gender	Male	117 (63.60%)	BMI	<18.5	7 (3.80%)
	Female	67 (36.40%)		18.5–23.9	68 (37.00%)
Age(years)	18~59	58 (31.50%)		24.0–27.9	79 (42.90%)
	60~74	76 (41.30%)		≥28	30 (16.30%)
	≥75	50 (27.20%)	Course of CAD	<6 months	65 (35.30%)
Educational level	Primary education or less	74 (40.20%)		≥6 months	119 (64.70%)
	Junior school	41 (22.30%)	Number of hospitalizations	1–3 times	145 (78.80%)
	High school or above	69 (37.50%)		>3 times	39 (21.20%)
Monthly income (RMB/yuan)	≤3000	95 (51.60%)	Number of stent implantations	0	84 (45.70%)
	3001~5000	56 (30.40%)		1	50 (27.20%)
	5001~10000	27 (14.70%)		≥2	50 (27.20%)
	>10000	6 (3.30%)	Number of comorbidities	0	19 (10.30%)
Marital status	Married	146 (79.30%)		1	93 (50.50%)
	Divorced/widowed	38 (20.70%)		2	50 (27.20%)
Residence	Cities or towns	104 (56.50%)		≥3	22 (12.00%)
	Rural	80 (43.50%)	History of acute cardiac infarction	Yes	69 (37.50%)
Living situation	Alone	17 (9.20%)		No	115 (62.50%)
	Living with spouse	107 (58.20%)	NYHA cardiac function classification	Ⅰ	82 (44.60%)
	Living with children	23 (12.50%)		Ⅱ	54 (29.30%)
	Living with spouse and children	35 (19.00%)		Ⅲ	38 (20.70%)
	Living with parents	2 (1.10%)		Ⅳ	10 (5.40%)

### The level of self-care ability, illness perception and HPB among patients with SCAD

The score of SC-CHDI, IPQ-R, and HPLP-Ⅱ are shown in Table 2. In the SC-CHDI scale, the scores for self-care maintenance, self-care management, and self-care confidence were 53.33 (46.67, 63.33), 61.11 (44.44, 66.67), and 50.00 (38.89, 66.67) respectively, all falling short of 70 points. This suggested a less than satisfactory performance in self-care maintenance, management, and self-assurance among individuals suffering from SCAD. The total score of HPLP-II scale was (111.89±16.891), which indicated that the level of HPB was at moderate.

**Table 2:** The level of self-care ability, illness perception and HPB (*n* = 184).

Project	Number of items	Score range	Min	Max	Score
SC-CHDI					
Self-care maintenance	10	0~100	13.33	80.00	53.33 (46.67, 63.33)
Self-care management	6	0~100	22.22	88.89	61.11 (44.44, 66.67)
Self-care confidence	6	0~100	11.11	88.89	50.00 (38.89, 66.67)
IPQ-R					
Illness duration	6	6~30	11.00	30.00	22.00 (18.00, 26.00)
Illness consequence	6	6~30	13.00	30.00	21.00 (18.00, 23.00)
Personal control	6	6~30	9.00	30.00	20.00 (18.00, 23.00)
Treatment control	5	5~25	11.00	23.00	18.00 (17.00, 18.00)
Illness coherence	5	5~25	5.00	24.00	15.00 (14.00, 18.00)
Cyclical timeline	4	4~20	8.00	19.00	14.00 (13.00, 15.00)
Emotional distress	6	6~30	8.00	29.00	20.00 (16.00, 23.00)
HPLP-Ⅱ	52	52~208	74.00	168.00	111.89±16.891

Note: SC-CHDI = Self-Care of Coronary Heart Disease Inventory, IPQ-R = Revised Illness Perception Questionnaire, and HPLP-Ⅱ = Health-Promoting Lifestyle Profile Ⅱ.

### Relationship between self-care ability, illness perception and HPB

The scores on each dimension of the SC-CHDI and IPQ-R scales were approximately normally distributed, and the total score of HPLP-II was normally distributed. Thus, a Pearson correlation analysis was selected to investigate the relationships between self-care ability, illness perception and HPB (Table 3). A significant association was found between HPB and different perspectives of self-care ability (self-care maintenance: r = 0.339, *P*<0.001; self-care management: r = 0.352, *P*<0.001; self-care confidence: r = 0.569, *P*<0.001). In the relationship between HPB and illness perception, HPB was positively correlated with personal control (r = 0.250, *P* = 0.001), treatment control (r = 0.302, *P*<0.001), and illness coherence (r = 0.367, *P*<0.001), and negatively correlated with illness consequence (r = -0.240, *P* = 0.001), cyclical timeline (r = -0.215, *P* = 0.003), and emotional distress (r = -0.326, *P*<0.001) dimensions. In the relationship between self-care ability and illness perception, both self-care maintenance and self-care management were positively associated with illness duration and illness coherence, and negatively associated with personal control; self-care confidence was positively associated with personal control and treatment control, and negatively associated with cyclical timeline.

**Table 3:** Relationship between self-care ability, illness perception and HPB (*n* = 184).

Project	1	2	3	4	5	6	7	8	9	10	11
1.Self-care maintenance	1										
2.Self-care management	0.348**	1									
3.Self-care confidence	0.206**	0.258**	1								
4.Illness duration	0.322**	0.212**	0.031	1							
5.Illness consequence	- 0.019	0.091	- 0.004	0.123	1						
6.Personal control	- 0.254**	- 0.183*	0.203**	- 0.192**	- 0.289**	1					
7.Treatment control	- 0.138	- 0.036	0.287**	- 0.240**	- 0.308**	0.608**	1				
8.Illness coherence	0.325**	0.264**	0.144	0.438**	- 0.096	0.112	0.033	1			
9.Cyclical timeline	0.011	0.065	- 0.280**	0.134	0.271**	- 0.210**	- 0.284**	0.092	1		
10.Emotional distress	- 0.106	- 0.079	- 0.055	- 0.194**	0.471**	- 0.107	- 0.147*	- 0.372**	0.234**	1	
11.HPLP-Ⅱ	0.339**	0.352**	0.569**	0.066	- 0.240**	0.250**	0.302**	0.367**	- 0.215**	- 0.326**	1

*P < 0.05

**P < 0.01.

### Hierarchical linear regression analysis

A hierarchical linear regression analysis was conducted, using the total score of HPLP-II as the dependent variable (S1 Table). Given that statistically significant differences were observed in HPLP-II scores among SCAD patients with varying educational level (*F* = 13.360, *P*<0.001), monthly income (*F* = 6.581, *P*<0.001), marital status (t = 1.996, *P* = 0.047), residence (t = 4.353, *P*<0.001), NYHA cardiac function (*F* = 4.793, *P* = 0.003), these variables were included into Model 1 as control variables. In the second step, three dimensions of self-care ability were incorporated into Model 2. In the third step, seven dimensions of illness perception were added to Model 3. The results showed that educational level, marital status, self-care maintenance, self-care management, self-care confidence, illness coherence, and emotional distress were potential influencing factors of HPB. There was no multicollinearity between the independent variables in this study.

### Mediating role of illness perception between self-care ability and HPB

**Fig 2:**
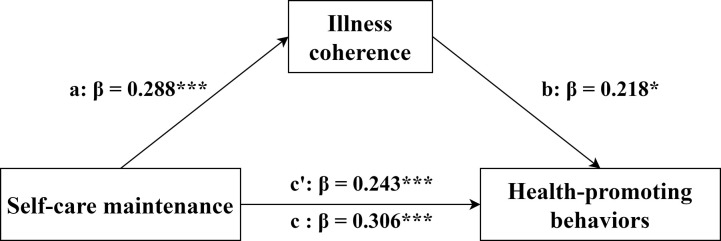
The model of illness coherence mediates the association between self-care maintenance and HPB. Note: c = total effect, c’ = direct effect, β = standardized coefficient, *P<0.05, ***P<0.001.

**Fig 3:**
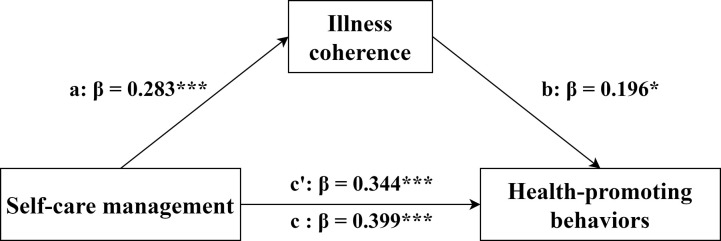
The model of illness coherence mediates the association between self-care management and HPB. Note: c = total effect, c’ = direct effect, β = standardized coefficient, *P<0.05, ***P<0.001.

We performed mediation effects model analysis in SPSS 25.0 using the PROCESS version 4.2 plug-in. We employed a bootstrapping procedure with 5,000 resamples and Model 4 to estimate indirect effects. Significant mediating (indirect) effects were established by excluding zero through 95% confidence intervals. Educational level, monthly income, marital status, residence, and NYHA cardiac function were set as covariates. The results presented in Table 4 and Fig 2 demonstrated that self-care maintenance (independent variable) exerted a significant positive influence on illness coherence (mediating variable) (*β* = 0.288, *P*<0.001) and HPB (dependent variable) (*β* = 0.306, *P*<0.001). Illness coherence also showed a significant positive impact on HPB (*β* = 0.218, *P* = 0.002). The total effect and direct effect of self-care maintenance on HPB were 0.306 and 0.243, respectively, with neither 95% Confidence Interval crossing zero. Consequently, it was evident that illness coherence exhibited a partial mediating effect between self-care maintenance and HPB (*β* = 0.063, 95% CI: 0.021~0.111), accounting for 20.59% of the total effect. Similarly, we concluded that illness coherence (mediating variable) also had a partial mediating effect (*β* = 0.055, 95% CI: 0.016~0.105) between self-care management (independent variable) and HPB (dependent variable), accounting for 13.78% of the total effect (Fig 3). However, none of the dimensions of illness perception mediated between self-care confidence and HPB, with a 95% CI after 0. Self-care confidence directly influenced HPB, accounting for 92.40% of the total effect.

**Table 4:** The mediating effect of illness perception between self-care ability and HPB in SCAD patients (*n* = 184).

Variables	Paths	Effect	SE	95% CI	
				LLCI	ULCI
Total effect	Self-care maintenance→HPB	0.306	0.066	0.175	0.437
	Self-care management→HPB	0.399	0.066	0.269	0.529
Direct effect	Self-care maintenance→HPB	0.243	0.068	0.109	0.377
	Self-care maintenance→Illness coherence	0.288	0.071	0.148	0.427
	Illness coherence→HPB	0.218	0.069	0.083	0.354
	Self-care management→HPB	0.344	0.067	0.212	0.477
	Self-care management→Illness coherence	0.283	0.073	0.138	0.427
	Illness coherence→HPB	0.196	0.066	0.065	0.327
Indirect effect	Self-care maintenance→Illness coherence→HPB	0.063	0.023	0.021	0.111
	Self-care management→Illness coherence→HPB	0.055	0.023	0.016	0.105

Note: HPB = health-promoting behaviors, Effect = standardized effect, SE = standard error, CI = confidence interval, LLCI = bootstrap lower limit of confidence interval, ULCI = bootstrap upper limit of confidence interval.

## Discussion

In this study, the HPLP-II scale scores were 111.89 ± 16.891. The level of health-promoting behaviors (HPB) of SCAD patients was regarded as moderate, indicating that SCAD patients still need to further improve their life behaviors. This is in line with the majority of scholars [29–31]. An increasing number of studies have shown that maintaining a healthy lifestyle and behavior plays an important role in controlling the risk of coronary heart disease [32].

Self-care maintenance and self-care management in SCAD patients had a positive effect on HPB. The results of mediation effect analysis in this study showed that the direct effect of self-care maintenance on HPB was 0.243, indicating that self-care maintenance has a positive effect on HPB. This can potentially be ascribed that patients with better treatment compliance and self-monitoring are more likely to spontaneously engage in HPB, actively undertaking disease management and cardiac rehabilitation. Self-care maintenance can reflect the patient’s autonomy in adhering to the treatment plan and self-monitoring, including timely follow-up, taking medication as prescribed by the doctor, adhering to exercise, reasonable diet and other behaviors [21]. Riegel proposed that the first step in the self-care process was maintenance, which included adherence to treatment behaviors [33]. Self-care management can reflect patients’ ability to recognize and cope with coronary symptoms. The third process of self-care is symptom management [33]. Poor self-care management of patients can lead to their readmission and prolonged hospital stay [34]. The results of mediation effect analysis in this study showed that the direct effect of self-care management on HPB was 0.344, indicating that self-care management also has a positive effect on HPB. The higher a patient’s self-management capability, the greater the frequency of HPB observed. Self-care maintenance and self-care management could jointly reflect the patient’s ability to self-care [22]. The study by Du et al., conducted in Yunnan, China, also revealed a positive correlation between self-care ability and HPLP-II scale scores [14]. And Chen HH [35] and Zhu FL [36] reached similar conclusions. The better engagement of HPB also means that patients will further have a higher quality of life [14]. Those with enhanced self-care capabilities are more likely to focus on their health, dietary choices, and physical activities, thereby fostering the health behaviors [14]. Nevertheless, within our research, it has been observed that the scores on the dimensions of self-care maintenance and self-care management remained disappointingly low. This underscored that the self-care ability among SCAD patients was still suboptimal, warranting further attention and intervention at the time. This phenomenon might be correlated with the predominantly older age and lower educational attainment of the majority of our study participants.

Illness coherence in SCAD patients has a positive effect on HPB. This study found that illness coherence had a positive effect on HPB. MacInnes also noted in his study on heart failure that illness coherence positively predicts patients’ self-care behaviors [37]. Kwon’s survey of patients with coronary artery disease similarly concluded that there was a positive correlation between illness perceptions and health behaviors [38]. The concept of illness coherence reflects the patient’s level of understanding of their condition. The higher a patient’s comprehension of their illness, the more likely they are to spontaneously engage in HPB.

There was a partial mediating effect of illness coherence between self-care maintenance and HPB. Self-care maintenance not only exerted a direct influence on HPB in patients with SCAD, but also indirectly impacted HPB through the coherence of their illness perception. The mediating value was 0.063, which constituted 20.59% of the total effect. It is plausible that patients with higher illness adherence have a deeper understanding of their condition, enabling them to proactively engage in HPB, thereby facilitating their physical recovery. Patients with better self-care maintenance abilities are likely to enhance their comprehension of the disease and develop a more positive perception of it through regular follow-ups, strict medication compliance, and weight management. Illness coherence also had a partial mediating effect between self-care management and HPB. The mediating effect value was 0.055, accounting for 13.78% of the total effect.

Several studies from China have demonstrated that illness perception as a mediator between other factors and HPB [39, 40]. Wang investigated patients with atrial fibrillation and found that illness perception was a mediating variable between cardiac rehabilitation and HPB [39]. Zhang surveyed patients with coronary artery disease and found that illness perception had a mediating effect between HPB and cardiac function [40]. A Korean study also noted that illness perception fully mediated the relationship between Type D personality and health behaviors, with illness perception significantly influencing patients’ health behaviors and clinical outcomes [38]. In previous studies, scholars from China, Jordan, Tanzania, and Iran have noted poor level of illness perception in patients with heart disease [34, 39, 41, 42]. The reasons for this may be as follows: 1) The patients themselves have not yet perceived serious symptoms and do not take the disease seriously. 2) Clinical nurses usually focus on basic nursing operations and prone to neglect customized health education. 3) The nurses’ own knowledge regarding self-care management of illnesses and their communication skills are suboptimal. 4) The patient’s level of comprehension regarding the nature of their ailment is deficient, impairing their ability to accurately grasp the progression and prognosis of the disease. 5) The provision of health education materials to patients is insufficient.

Furthermore, some studies have shown that illness perception has an important impact on the improvement of self-care ability and the practice of HPB [43, 44]. Patients exhibiting more pessimistic illness perceptions are prone to adopting maladaptive coping strategies and manifesting lower levels of self-care proficiency. Conversely, individuals harboring positive perceptions regarding their condition are inclined to-wards proactive learning and exhibit higher levels of self-care competence [44]. Meanwhile, enhanced self-care skills empower patients to effectively acquire pertinent disease-related information and bolster their motivation to embrace and sustain HPB. As patients recognize the advantages of self-care, their outlook towards their illness becomes more optimistic, prompting a greater inclination towards adopting healthier behaviors [35, 45]. In general, patients with higher levels of self-care ability are more likely to actively seek external support, possess positive illness perception, and maintain a positive attitude. Consequently, they are better equipped to proactively engage in treatment adherence and implement HPB. The American Heart Association’s Scientific Statement for Healthcare Professionals stated that self-care maintenance requires individuals to maintain physical and emotional stability [12]. Self-care maintenance encompasses sustained cardiovascular health behaviors, acquisition of knowledge, and adherence to condition-specific treatments. In previous studies, most nurse-led interventions for patients’ self-care maintenance and management at home have taken the form of curriculum-based education and motivational interviewing [46]. Self-care management includes the patient’s recognition of cardiovascular disease symptoms, and the measures taken to cope with the symptoms. Nurse-led self-care interventions significantly improved patients’ self-care maintenance compared to usual care. Over the course of a year, patients have approximately only ten hours of engagement with healthcare providers within hospital settings [12]. The self-care maintenance and self-care management of patients are predominantly carried out by the patients themselves and their families in settings outside of the hospital. This underscores the pivotal role of community workers. For SCAD patients, there is a heightened need to understand how to adapt to specific dietary recommendations [35]. With the advancement of technology and the widespread adoption of the internet, virtual reality devices, real-time monitoring equipment, remote rehabilitation platforms, and applications have been integrated into health education and rehabilitation for patients with coronary heart disease [47].

This study, along with previous research [48], has underscored the significant correlation between illness perception and HPB. Patients with a clear understanding of their illness are more likely to engage in proactive HPB aimed at managing their condition. This highlights the imperative for healthcare professionals to elucidate the etiology of the disease, clinical manifestations, treatment modalities, and prognoses to patients, thereby steering them towards cultivating accurate perceptions of their condition. Furthermore, it is essential for medical practitioners to employ various strategies to cultivate a positive illness perception among patients. This can be achieved through the use of encouraging language, sharing success stories of rehabilitation cases, and providing rehabilitation diaries [34, 49]. These approaches serve to incentivize patients to actively acquire self-care knowledge and embrace HPB autonomously. By fostering a constructive perception of their illness, individuals are more likely to proactively engage in activities that contribute to their overall well-being. In addition, to increase the motivation of SCAD patients to actively participate in HPB, a multi-party collaboration among hospital healthcare workers, community workers, and patients’ family members is also needed.

## Conclusion

Within this study, the findings identified three dimensions of self-care ability (self-care maintenance, self-care management, self-care confidence), two dimensions of illness perception (illness coherence, emotional distress) were the influencing factors of HPB. Illness coherence partially mediated the effect between self-care maintenance and HPB. Similarly, illness coherence partially mediated the effect between self-care management and HPB. In the future, it is necessary for hospital healthcare workers, community workers, and patients’ families to work together to focus on the self-care ability and positive illness perception of patients with cardiovascular disease, so as to increase patients’ motivation to participate in HPB and improve their quality of life.

## Supporting information

S1 Table Hierarchical linear regression analysis of HPB of SCAD patients. (PDF)
