# Supplementary material for: Mediating effect of illness perception between self-care ability and health-promoting behaviors among patients with stable coronary artery disease
Source: PLoS One. 2025 Feb 21;20(2):e0316551. doi: 10.1371/journal.pone.0316551 (PMC11845029; doi:10.1371/journal.pone.0316551)
Supplement: S1 Table — (PDF) [file pone.0316551.s001.pdf]

S1 Table. Hierarchical linear regression analysis of HPB of SCAD patients (*n*=184)

Variables	Model I			Model II			Model III		
	β	t	*P*	*β*	t	*P*	*β*	t	*P*
Educational level, Junior school (reference: Primary education or less)	0.060	0.701	0.484	0.119	1.777	0.077	0.139	2.110	0.036
Educational level, High school or above (reference: Primary education or less)	0.266	2.513	0.013	0.179	2.168	0.032	0.188	2.336	0.021
Monthly income, 3000~5000 yuan (reference:<3000 yuan)	0.013	0.142	0.887	-0.064	-0.865	0.388	-0.100	-1.385	0.168
Monthly income, 5001~8000 yuan (reference:<3000 yuan)	0.034	0.365	0.715	-0.006	-0.088	0.930	-0.051	-0.701	0.484
Monthly income, >10000 yuan (reference:<3000 yuan)	0.047	0.608	0.544	0.108	1.796	0.074	0.059	0.991	0.323
Marital status	0.103	1.451	0.148	0.102	1.846	0.067	0.110	1.990	0.048
Residence	-0.124	-1.418	0.158	-0.058	-0.837	0.404	-0.040	-0.604	0.546
NYHA cardiac function, II class (reference: I class)	0.105	1.366	0.174	0.015	0.254	0.800	0.057	0.966	0.335
NYHA cardiac function, III class (reference: I class)	-0.118	-1.492	0.137	-0.159	-2.521	0.013	-0.066	-0.971	0.333
NYHA cardiac function, IV class (reference: I class)	-0.049	-0.670	0.504	-0.136	-2.341	0.020	-0.058	-0.948	0.344
Self-care maintenance				0.159	2.686	0.008	0.172	2.799	0.006
Self-care management				0.236	3.782	<0.001	0.204	3.358	0.001
Self-care confidence				0.435	7.500	<0.001	0.387	6.385	<0.001
Illness duration							-0.082	-1.351	0.179
Illness consequence							-0.046	-0.641	0.523
Personal control							0.023	0.302	0.763
Treatment control							0.092	1.310	0.192
Illness coherence							0.142	2.152	0.033
Cyclical timeline							-0.044	-0.746	0.457
Emotional distress							-0.137	-2.111	0.036
F value		4.363			14.606			11.868	
R^2		0.201			0.528			0.593	
Adjusted R^2		0.155			0.491			0.543	

Note: *β*=standardized beta.
